# Efficacy and safety of ginkgo preparation in patients with vascular dementia: A protocol for systematic review and meta-analysis

**DOI:** 10.1097/MD.0000000000022209

**Published:** 2020-09-11

**Authors:** Miyuan Wang, Hongye Peng, Zexu Peng, Kailin Huang, Tingting Li, Lei Li, Xin Wu, Heyuan Shi

**Affiliations:** a Beijing University of Chinese Medicine, Beijing; b Hubei University of Chinese Medicine, Wuhan Hubei, China.

**Keywords:** ginkgo preparation, meta-analysis, protocol, systematic review, vascular dementia

## Abstract

**Background:**

Vascular dementia has become the second most common type of dementia after Alzheimer disease. At present, there is no uniform standard for VaD treatment guidelines among countries. The efficacy of ginkgo biloba in the treatment of vascular dementia is still controversial. The purpose of this study is to evaluate the effectiveness and safety of ginkgo biloba in the treatment of vascular dementia through meta-analysis.

**Methods:**

Six English databases (PubMed, Web of science, Medline, EBASE, Springer Cochrane Library, and WHO International Clinical Trials Registry Platform) and 4 Chinese databases (Wan fang Database, Chinese Scientific Journal Database, China National Knowledge Infrastructure Database(CNKI) and Chinese Biomedical Literature Database) will be searched normatively according to the rule of each database from the inception to August 1, 2020. Two reviewers will independently conduct article selection, data collection, and risk of bias evaluation. Any disagreement will be resolved by discussion with the third reviewer. Either the fixed-effects or random-effects model will be used for data synthesis based on the heterogeneity test. The change in the scores on mini-mental state examination, activity of daily living scale and Montreal cognitive assement will be used as the main outcome measure, Hamilton depression scale, Hastgawa dementia scale, blessed dementia scale, clinical dmentia rating scale as the secondary outcome. Treatment emergent symptom scale, general physical examination (temperature, pulse, respiration, blood pressure), Routine examination of blood, urine and stool, electrocardiogram, liver and kidney function examination as the security indexs. RevMan5.3.5 will be used for meta-analysis.

**Results:**

This study will provide high-quality evidence to assess the effectiveness and safety of ginkgo preparation for vascular dementia.

**Conclusion:**

This systematic review will explore whether ginkgo preparation is an effective and safe intervention for vascular dementia.

**Ethics and dissemination:**

Ethical approval are not required for this study. The systematic review will be published in a peer-reviewed journal, presented at conferences, and will be shared on social media platforms. This review will be disseminated in a peer-reviewed journal or conference presentation.

**PROSPERO registration number:**

PROSPERO CRD42020167851.

## 1 Introduction

Vascular dementia is a type of vascular cognitive impairment which finds its way to become the second most common type of dementia after Alzheimer disease.^[1,2]^ Brain small vascular dementia, a vascular disease, is the predominant factor contributing 45% of dementia.^[3]^ The main clinical manifestations demonstrated are cognitive, psychological, and behavioral symptoms, such as repetitive questioning, restlessness, depression, apathy, confusion, aggression, sleep disorders, and various misbehaviors.^[4]^ In 2018, Alzheimer disease international had estimated a total of 50 million people are suffering from dementia. This figure is believed to increase exponentially and reach 82 million people by the time of 2030. One hundred fifty-two million of the world population will be suffering from dementia by 2050 in regard of the estimation.^[5]^ The behavioral and psychological symptoms caused by dementia bring serious distress to the patients and their nursing staff while burdening social economy all along. According to the Alzheimer disease international, 16 million family members and other volunteered caretaker in the United States of America had provided a total of 18.6 billion hours of caretaking for dementia patients during 2019. The cost alone reached an approximation of 244 billion United States dollar. It is further estimated that, 305 billion United States dollar will be involved by the time of 2020 to cover the healthcare, long-term nursing care, and hospice care expenses for elder dementia patients aged 65 or above.^[6]^

There is currently no uniformed standard on the treatment of dementia. Commonly acknowledged therapeutic drugs include cholinesterase inhibitors (such as donepezil, galantamine, and NMDA receptor antagonists such as Memantine),^[7]^ etc, which are proved to improve the cognitive function and scoring a higher behavior scale on patients to varying degrees.^[8–10]^ However, these drugs will produce various adverse effects, such as digestive tract reactions, joint pain, dizziness and headache, constipation, etc.^[11–13]^ Its ability to provide overall improvements on patients is also yet to be determined.^[11]^

Ginkgo biloba extract has been reported as a natural medicine since the 1980s,^[14]^ after that, many clinical studies had been conducted. Researches have proven ginkgo biloba extract ability to improve cognitive function, daily activities, and the equality of life in patients with VD regardless of the severity of their neuropsychiatric symptoms, while demonstrating good tolerance.^[15–17]^ Hashiguchi et al had systematically reviewed the relevant literature prior to 2014. A conclusion was drawn that ginkgo biloba extract EGb 761 240 mg per day is safer for patients with mild to moderate vascular dementia an demonstrated a better treatment effect in comparison to placebo.^[18,19]^ Li et al discovered through clinical trials that ginkgo biloba improves blood lipids, lipoproteins, hemorheological parameters, and cognitive function in patients with mild vascular cognitive impairment.^[20]^

However, there is still a lack of meta-analysis on ginkgo biloba extract compared with conventional western medicine treatment, and ginkgo biloba extract combined with western medicine for vascular dementia treatment. Besides, the publications of some late clinical trials also prompted us to conduct a systematic review and meta-analysis of the existing randomized controlled trials to evaluate the efficacy and safety of ginkgo biloba extract.

## 2 Methods

### 2.1 Study registration

The protocol of this review has been registered in the International Prospective Register Of Systematic Reviews (PROSPERO; http://www.crd.york.ac.uk/PROSP-ERO/display_record.php?ID=CRD42020167851). It was reported following the statement guidelines of preferred reporting items for systematic reviews and meta-analyses protocols.^[21]^

### 2.2 Inclusion criteria for study selection

#### 2.2.1 Types of studies

Randomized controlled trials (RCTs) on ginkgo preparation for vascular dementia published in Chinese and English will be included to ensure the quality of this systematic review. The current clinical trial results will be integrated objectively to evaluate the efficacy and safety of ginkgo preparation in the treatment of vascular dementia.

Non-RCTs, quasi-RCTs, cohort studies, reviews, case reports, experimental studies, expert experience, the data of the included study is missing or incomplete, and duplicate publications will be excluded to ensure the quality of this systematic review.

#### 2.2.2 Types of participants

Patients with a clinical diagnosis of vascular dementia, regardless of nationality, race, gender, occupation, and educational background will be considered. While the cause of vascular dementia is not limited, all patients should be diagnosed as VD in accordance to at least one of the current or past definitions or guidelines of VD, such as:

1. Diagnostic and Statistical Manual of Mental Disorder 4th, DSM-IV.
2. National Institute of Neurological Disease and Stroke— Association Internationale pour la Rechercheer l”Enseignement et Neurosciences, NINDS-AIREN.
3. International Classification of Disease, ICD-10-R.
4. Alzheimer Disease Diagnosis and Treatment Center, ADDTC.

#### 2.2.3 Types of interventions

This study focuses on the clinical trial (RCTs) of vascular dementia treated under a combination treatment of ginkgo preparation and western medicines. The results are anticipated to aid clinicians. All trials with an assessment of the combination treatment mentioned above will be included, while studies of control group could only use western medicines as the sole treatment.

#### 2.2.4 Types of outcome measures

##### 2.2.4.1 Primary outcomes

The primary outcomes are mini-mental state examination, activity of daily living scale and Montreal cognitive assement.

##### 2.2.4.2 Secondary outcomes

The secondary outcomes of this review mainly include the following aspects:

1. Hamilton depression scale.
2. Hastgawa dementia Scale.
3. Blessed dementia scale.
4. Clinical dmentia rating Scale

##### 2.2.4.3 Security Index

1. Treatment Emergent Symptom Scale.
2. General physical examination (temperature, pulse, respiration, blood pressure).
3. Routine examination of blood, urine and stool.
4. Electrocardiogram.
5. Liver and kidney function examination.

### 2.3 Data sources

Literature in the following databases will be retrieved from the inception to August 1, 2020: 6 English databases (PubMed, Web of science, Medline, EBASE, Springer Cochrane Library, and WHO International Clinical Trials Registry Platform) and 4 Chinese databases (Wanfang Database, Chinese Scientific Journal Database, China National Knowledge Infrastructure Database, and Chinese Biomedical Literature Database).

### 2.4 Searching strategy

Search strategy will be built in accordance to the guidelines from the Cochrane handbook. The details of the search strategy for PubMed is presented in Table 1 including all the search terms, while similar search strategies will be applied for all electronic databases.

**Table 1:**
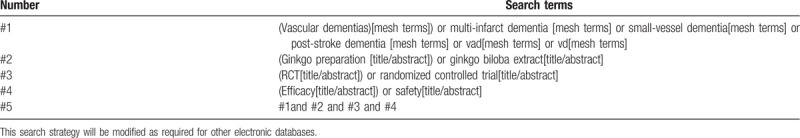
PubMed Search Strategy.

### 2.5 Data collection and analysis

#### 2.5.1 Selection of studies

**Figure 1:**
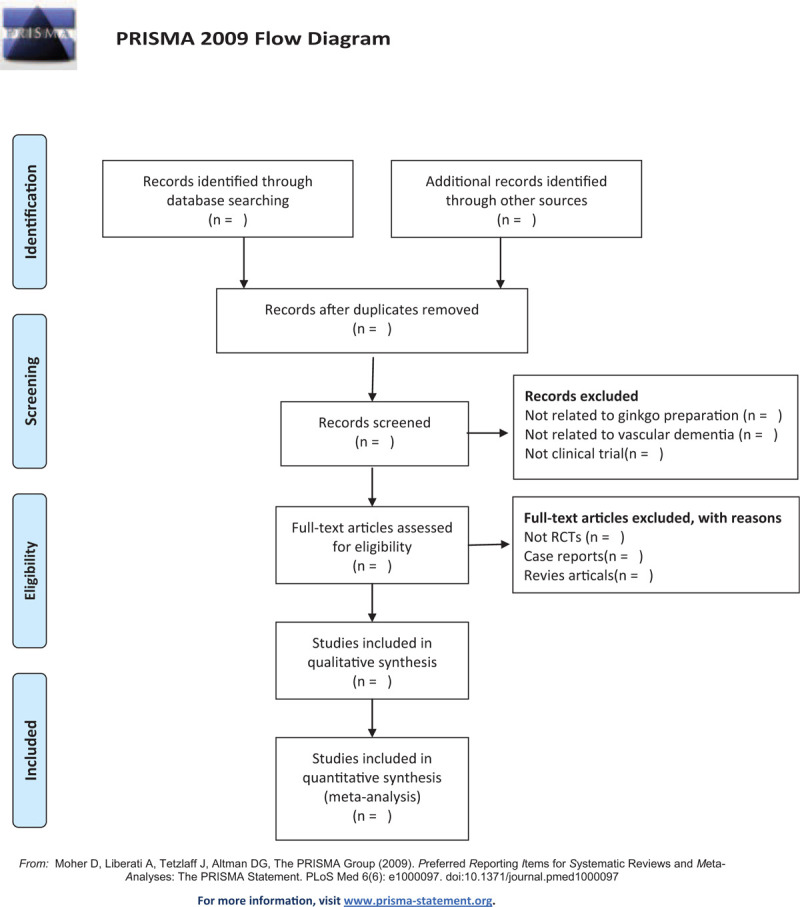
The PRISMA flow chart of the selection process.

The basic process of literature inclusion will be pursued with reference to the Cochrane Collaboration System Evaluator Manual (5.1.0). Relevant literatures will be obtained from specified databases, later imported into a database created by Endnote X7. Duplicate documents will be screened out through this process. Independent screening of titles, abstracts, and keywords of all retrieved records will be performed by 2 researchers. The following items will be included in the data extraction table: title, first author, publishing year, country, database, and design of the study. The authors of a study failed to construct clear and adequate information will be contacted via email. Reasons of inclusion and exclusion (PICOS) will be disclosed in a spreadsheet during abstract screening and full-text evaluation. The screening flow diagrams of this study will be shown in Figure 1.

#### 2.5.2 Data extraction and management

Two independent reviewers will extract the data of interest from the eligible study and fill in the data collection sheet. Any disagreement will be resolved by consensus or consultation with the third reviewer. Microsoft Excel 2013 will be used for data and information management. We will extract the following data:

1. The basic characteristics of RCT: title, 1st author, publishing year, country, and the journal.
2. Participants’ characteristics: average age, gender, sample size, inclusion and exclusion criteria, baseline situation, type, follow-up time points.
3. Interventions: treatment duration, study design, randomization, allocation concealment, and blinding methods
4. Comparators: western medicines
5. Outcomes: measures, primary and secondary outcomes, security indexes.

#### 2.5.3 Assessment of risk of bias

The risk of bias of the selected RCTs will be accessed by 2 reviewers separately using the Cochrane bias risk tool (RevMan5.3.5). This tool has the following 6 domains: random sequence generation, allocation concealment, blinding, incomplete outcome data, selective reporting, and other bias. Each potential trial of bias will be graded as high, low, or unclear. When 2 quality reviewers are unable to reach a consensus through negotiation on risk assessment, the third the third reviewer will make the decision.

#### 2.5.4 Measures of treatment effect

Mean differences (MD) or standard mean difference (SMD) with 95% CIs will be used as continuous data, and the dichotomous outcomes will be estimated by the risk ratio (RR) with 95% confidence intervals (CIs).

#### 2.5.5 Unit of analysis issues

Only the 1st experimental period data of crossover trials will be extracted to minimize carryover effects. For trials regarding multiple interventions, all relevant experimental groups and control groups within the trial will be combined into a single group to avoid unit-of-analysis error.

#### 2.5.6 Management of missing data

The original author would be enquired for missing data. If the data fail to be provided upon request, it will be excluded from the study.

#### 2.5.7 Assessment of heterogeneity

Heterogeneity will be assessed by visual inspection of the forest plots and detected by standard χ^2^ test and I^2^ test. It will be considered as no significant heterogeneity between the trials when *P* > .1, I^2^ < 50%, the fixed effect model will be applied for statistics. Otherwise, the random effect model will be chosen. Sensitivity analysis or meta regression will be performed when heterogeneity occurs to assess the source.

#### 2.5.8 Assessment of reporting biases

If 10 or more studies are included in the meta-analysis, funnel plots will be used to evaluate the reporting bias. Egger and Begg tests are further applied to conduct quantitative evaluation of publication bias. The trim and fill method will be used to identify and correct asymmetric funnel arising from publication bias if appropriate.^[22]^

#### 2.5.9 Data synthesis

If studies are adequately homogeneous in design and comparison, data synthesis using Review Manager 5.3.5 Software will be conducted.^[23]^ Forest plots and heterogeneity tests between the included studies will be obtained by the software. Continuous data will be expressed as MD/SMD with 95% CIs, while the dichotomous outcomes will be presented as RR/OR with 95% CIs.

#### 2.5.10 Subgroup analysis

Subgroup analysis will be applied when heterogeneity is detected (e.g., different types of western medicines therapies, patient conditions, research quality and publication year) to spot the source of heterogeneity.

#### 2.5.11 Sensitivity analysis

Sensitivity analysis will be based on heterogeneity, while the robustness and reliability of merged outcome results will be examined with the exclusion of small and low-quality studies.

## 3 Discussion

Chinese herbal medicine has thousands of years of history and is now widely applied in the treatment of many diseases, including dementia. Studies have proven that medicinal plants can improve behavioral and psychological symptoms, working memory, daily activities, and neuropsychiatric inventories. Traditional Chinese medicine treatment may therefore be a potential treatment method for VaD.^[24]^ Ginkgo biloba, as natural herb, possesses the effects of activating blood and removing blood stasis, dredge collaterals to relieve pain, restrain lungs to relieve coughing. The main components of its extract are flavonoids and terpenoid trilactones.^[25]^ Such substances are widely used in ischemic cerebrovascular diseases, vascular dementia, Alzheimer disease, liver cancer, and glaucoma etc.^[26–29]^ While as a natural medicine, ginkgo biloba extract is a multi-target, multi-channel drug in possess of a large potential. There is currently no uniformed standard or guidelines for VaD treatment among countries. Although many clinical studies have confirmed that ginkgo biloba extract can effectively enhances behavioral, psychological symptoms, and cognitive level of VaD patients,^[30]^ its efficacy and safety remained controversial.

The purpose of this study is to systematically review and evaluate all randomized controlled trials of ginkgo biloba extract compared with conventional western medicine treatment, and ginkgo biloba extract combined with western medicine in the treatment of vascular dementia. This systematic review and meta-analysis will provide a convincing conclusion on the effectiveness and safety of ginkgo biloba extract combined with western medicine in the treatment of vascular dementia. In addition, this study will aid clinician on vascular dementia treatment, benefits the corresponding patients, and provides reliable references for a wide application.

## Author contributions

MYW, HYP, ZXP, and HYS conceived and designed the protocol, and MYW drafted the protocol manuscript. HYP developed the search strategy, with input from KLH and TTL. MYW, HYP and LL planned the data extraction. MYW, HYP and XW planned the quality appraisal of all included studies. MYW, HYP, ZXP, KLH, TTL, LL, XW, and HYS, critically revised the manuscript for methodological and intellectual content. All authors approved the final version.

**Conceptualization:** Miyuan Wang, Hongye Peng, Zexu Peng, Heyuan Shi.

**Data curation:** Zexu Peng, Tingting Li, Lei Li, Kailin Huang.

**Formal analysis:** Miyuan Wang, Xin Wu.

**Project administration:** Miyuan Wang, Hongye Peng, Zexu Peng.

**Supervision:** Miyuan Wang, Heyuan Shi, Hongye Peng, Zexu Peng.

**Writing – original draft:** Miyuan Wang, Hongye Peng, Zexu Peng.

**Writing – review & editing:** Miyuan Wang, Heyuan Shi.
